# Prediction of gestational diabetes mellitus in the first trimester: comparison of maternal fetuin-A, N-terminal proatrial natriuretic peptide, high-sensitivity C-reactive protein, and fasting glucose levels

**DOI:** 10.20945/2359-3997000000203

**Published:** 2020-03-04

DOI: 10.20945/2359-3997000000126

Arch Endocrinol Metab. 2019;63(2):121-7

Where you read:

Hatice Kansu-Celik^1^, A. Seval Ozgu-Erdinc^1^, Burcu Kisa^1^, Rahime Bedir Findik^1^, Canan Yilmaz^1^, Yasemin Tasci^1^

^1^ University of Health Sciences, Zekai Tahir Burak Health Practice Research Center, Ankara, Turkey

Should read:

Hatice Kansu-Celik^1^, A. Seval Ozgu-Erdinc^1^, Burcu Kisa^1^, Rahime Bedir Findik^1^, Canan Yilmaz^2^, Yasemin Tasci^1^

^1^ University of Health Sciences, Zekai Tahir Burak Health Practice Research Center, Ankara, Turkey

^**2**^ **Gazi University Faculty of Medicine, Department of Medical Biochemistry, Ankara, Turkey**

